# Production of gelatin fibrous mats using different nanofiber production methods for medical applications and comparison of their properties

**DOI:** 10.55730/1300-0527.3631

**Published:** 2023-10-16

**Authors:** Melike GÜNGÖR, Kevser SAĞLAMKOL, Zeynep Yağmur BAYDEMİR, Ali KILIÇ

**Affiliations:** 1 TEMAG Laboratories, Faculty of Textile Technologies and Design, İstanbul Technical University, İstanbul, Turkiye; 2 Areka Group LLC, İstanbul, Turkiye

**Keywords:** Nanofibers, gelatin, nanofiber production methods, medical applications

## Abstract

In the literature, there are studies on medical applications using different nanofiber production methods with natural polymers. However, each system has different fiber-forming capabilities. For this reason, in this study, we investigated the production of nanofibers from a biodegradable natural polymer, gelatin, using four separate nanofiber production methods, namely electrospinning (ES), electroblowing (EB), solution blowing (SB), and centrifugal spinning (CS). Our aim was to determine the most suitable fibrous web structure for medical applications and contribute to science in this respect. It was observed that the thinnest fibers (386 nm) and the heaviest mats (15.975 g m^−2^) were produced by the SB method as a result of using 10 wt.% gelatin solution with a total of 10 mL. With the ES and EB methods, tighter fabric structures were obtained than with the others due to the presence of electric fields. In the CS method, more and bead-free fibers were produced due to the increase in viscosity with a 12.5 wt.% gelatin solution. Moreover, with the concentration of 12.5 wt.%, the fiber diameters of SB and CS samples increased about 2-fold.

## 1. Introduction

With the advancement in technology, the utilization of nano- and microfiber products in the medical field has significantly increased. Synthetic or natural polymers are used to create man-made fibers. Natural polymers, such as cellulose, collagen, and gelatin, are used in the medical field due to their biocompatibility. Gelatin, obtained from the hydrolysis of thermally denatured collagen, is a biomaterial that is nontoxic, biodegradable, and nonimmunogenic [1–4]. Gelatin is widely used in biomedical applications, including controlled drug release vehicles and wound dressings due to its appropriate absorption efficiency. In addition, it can mimic the extracellular matrix [3] and some of its amino acids facilitate wound healing and tissue regeneration [5]. Gelatin has a highly hydrophilic structure and good gas permeability [4]. At the same time, it is a cost-effective material from an industrial point of view [1,3]. Due to these advantages, gelatin has been widely studied in the field of nanofibers.

Various methods are used to produce nanofibers, including electrospinning (ES), electroblowing (EB), solution blowing (SB), and centrifugal spinning (CS). The ES method is one of the most common and oldest methods used to produce nanofiber structures. The ES system consists of a syringe pump, syringe, collector plate or rotating drum, and a power supply. When the system is started, the needle or nozzle tip, which is negatively charged by the applied voltage, induces the solution and causes instability in the solution. The creation of electrostatic forces between the anode and the cathode overcomes the surface tension, and the solution slowly thins, forming a Taylor cone [6,7]. With the removal of the solvent, the solution turns into a fiber form, which is collected on the positively charged collector [8–11].

In the SB method, fiber formation occurs with compressed air. The polymer solution fed to a needle or nozzle tip is hit with high air pressure, quickly removing the solvent [12] and the elongated solution jet is collected on a vacuum collector in the form of fibers [13]. It is possible to produce nanosized fibers with this method without the use of high-voltage electric fields. In contrast to the ES method, the SB method is considered more reliable because of the absence of an electric field, and nozzles can be easily interfered with during production.

The EB method involves forming nanofibers through the combined effect of compressed air and an electric field [14]. While the solution fed to the nozzle tip is converted into fiber form with high air pressure, the solution that cannot be unstable during production and cannot change to fiber form is charged with electricity by the power supply connected to the nozzle tip and it is transferred to fiber form.

The CS method, which is a nanofiber production system that employs centrifugal forces, is an old production method that was first used to produce glass wool in micron diameters. However, it is considered new for nanofiber production [15]. In this system, a viscoelastic solution fed into the spinning nozzle or plate overcomes the surface tension and moves away from the nozzle by rapid rotation [16]. The evaporation of the solvent in the resulting solution jet is collected on the collector in the form of fibers. Compared to other nanofiber production systems, particularly the ES method, this system is more reliable [15,17]. With this production method, more aligned and high polymer chain orientation fibers can be produced at high production speeds up to 100–200 times [17,18]; thus, CS is a cost-effective and easy-to-use method [19]. The advantages and disadvantages of the four nanofiber production methods are given in Table 1.

### 1.1. Gelatin fiber production studies with the nanofiber production methods

In the study by Ki et al. [26], gelatin solutions were prepared using the ES method with concentrations of 8, 10, and 12 wt.% in a mixture of formic acid and water. The aim in the study was to examine the properties of the fibrous mats produced, with the collector–nozzle distance and electric field (6–25 kV) as system parameters. The production was carried out at room temperature and 60% relative humidity. The results showed that the electric field did not significantly affect fiber diameters but did affect the fiber distribution.

In another study, Panthi et al. produced nylon-6(PA6)/gelatin composite nanofibers using the ES method for use in biomedical applications. Gelatin solutions with concentrations of 6, 10, 20, and 30 wt.% were mixed with a 22 wt.% concentrated PA6 solution at a ratio of 4:1. The collector distance was kept constant at 15 cm and the electric field was 22 kV. The study showed that the gelatin additive did not affect the mechanical properties of PA6. Nanonets of about 10 nm were obtained from the PA6 solution mixed with 10 wt.% gelatin solution, which showed higher mechanical values. Production with high gelatin content (30 wt.%) showed better bioactivity [27].

Using the SB method, Vilches et al. carried out fiber production with fish gelatin. In order to examine the effect of fiber diameter on water absorption, a solution was prepared by dissolving gelatin at 3 different concentrations (15, 20, 25 wt.%) in acetic acid–water mixed solvent. The system parameters used in the production were 25 G needle diameter, 8.1 mL h^−^^1^ solution feed rate, 200 kPa air pressure, 70 rpm collector speed, and 74 mm cylindrical collector diameter. As a result of the production, fibers with a cylindrical and smooth structure were obtained, and the fiber diameters increased (~280 nm) with an increase in concentration. Coarse fiber gelatin mats showed higher water absorption capacity [28].

Singh et al. used the SB method to produce gelatin solutions with concentrations ranging from 10 wt.% to 45 wt.% using porcine gelatin (type A) and acetic acid. The aim of their study was to examine the effects of gelatin concentration and system parameters on fibers and the suitability of these fibers for tissue engineering applications. The system parameters included 50–350 kPa air pressure and 2–2000 μL min^−^^1^ solution feed rate. Thick diameter fibers (1 μm) were obtained with 20 wt.% and 35 wt.% solutions without droplets. Production was conducted at different air pressures with a 25 wt.% concentrated solution and finer fibers up to 100 nm were obtained with increasing air pressure. As a result, in in-vitro tests with hBMSC cells, it has been proven that the mats produced are biocompatible [29].

There are nanofiber production studies carried out by combining gelatin with other polymers using nanofiber production methods. Lordhuswamy et al. produced gelatin and polycaprolactone (PCL) nanofibers using the CS method. The aim of their study was biocompatible mat production. The gelatin used in the study was obtained from cattle. PCL/gelatin solutions with 15 wt.% solution concentration were prepared at the ratios of 100/0, 70/30, 50/50, and 30/70 and all production took place using 5000 rpm nozzle rotation speeds. Smooth, thin, and aligned fibers were obtained with the CS method. The presence of gelatin resulted in thinner fiber diameters and more porous mats, leading to higher absorbency and better wound healing possibility. In in-vivo tests, it was observed that the healing process in the rat group treated using gelatin mats was much faster. At the same time, it was concluded that the strength value of the leather piece treated with the PCL/gelatin mat was much higher than that of the control sample [30].

In a similar study conducted by Badrossamay et al., fibers made of PCL, PCL–collagen (75/25) and PCL–gelatin (75/25) were produced using the CS method. The purpose of their study was to demonstrate that the CS method results in higher protein adhesion capacity on the surface of fibrous mats and higher production speed for in-vitro and in-vivo applications of the fibers produced using this method. According to that study, the fibrous mats produced by the CS method exhibited anisotropic mechanical properties that closely resemble those of biological tissue. The study also showed that increasing the gelatin or collagen ratio led to an increase in solution viscosity and fiber diameters, but resulted in a less aligned fiber structure [31].

In our study the aim was to compare the old and new methods of producing fibrous mats using natural polymers, and to determine which method is most suitable for this purpose. Gelatin mats were produced using four different nanofiber production methods, and the basic properties of each mat were characterized. Initially, the solution concentration was kept constant at 10 wt.%, and the four different production methods were carried out while keeping the needle diameter, collector speed, and amount of solution consumed constant. Subsequently, the effect of solution concentration was observed by carrying out production in both SB and CS systems using a solution with a 12.5 wt.% concentration.

SEM images of each nanofibrous mat were obtained. Fiber diameters were measured on 50 different fibers using the SEM images. Since breathability is an important factor in medical applications, the air permeability of each mat was measured according to the EN ISO 9237 special standard. The air permeability value is important in establishing a relationship with the porosity of the structure. Finally, the solidities of each sample were calculated.

## 2. Materials and methods

### 2.1. Materials

Type B gelatin (Bloom 200–220) produced from bovine skin was purchased in powder form from Halavet Gida LLC (İstanbul, Türkiye). Glacial acetic acid was obtained from Merck (anhydrous, 100% purity) and used as a solvent.

### 2.2. Production of gelatin nanofibers with nanofiber production methods

Gelatin solutions with 10 wt.% and 12.5 wt.% solution concentrations were prepared for the production. A 22 G diameter needle was used in all systems and the production amount was adjusted to consume 10 mL of solution in total. The collector rotation speed was 1000 rpm. An Aerospinner L1.0 nanofiber production machine (AREKA Advanced Technologies Ltd. Comp., Türkiye) was used for ES, SB and EB production and a Nanocentrino L1.0 nanofiber production machine (AREKA Advanced Technologies Ltd. Comp., Türkiye) was used for CS production. Production parameters for ES: 30 kV electricity, 1 mL h^−^^1^ solution feed rate; production parameters for SB: 3 bar air pressure, 5 mL h^−^^1^ solution feed rate; production parameters for EB: 30 kV electricity, 3 bar air pressure, 5 mL h^−^^1^ solution feed rate; production parameters for CS: 8000 rpm nozzle rotation speed, 30 mL h^−^^1^ solution feed rate. The distance between the nozzle and the collector was 35 cm in the other 3 methods (ES, SB, and EB). All parameters are given in Table 2.

## 3. Characterization

### 3.1. Viscosity

The viscosity of all the gelatin solutions prepared was measured by viscometer (Rotational Viscometer, Fungilab, Alpha Series).

### 3.2. Morphology of the gelatin mats

Fiber morphologies of the fibrous mats were investigated by scanning electron microscopy (SEM) (Tescan Vega 3). Prior to SEM, the samples were sputter coated with a 10-nm thick gold/palladium (Au/Pd) layer to obtain a conductive layer on the surface. In addition, the program ImageJ was used to determine fiber average diameter of the samples. Images were taken at 500× and 5000× magnifications.

### 3.3. Air permeability of the samples

The air permeability of samples was measured using an air permeability device (Prowhite Airtest II) according to standard EN ISO 9237 in an area of 20 cm^2^ at 100 Pa pressure. Four measurements were made for each sample and the mean was taken.

### 3.4. Solidities of the samples

Solidity refers to the solid density in a sample with a certain area. Higher solidity means lower porosity. Solidity, also called fiber packing density, is calculated using Equation (1) [32]. The thickness of the nanofibrous mats was measured using a digital micrometer. The density of the polymer and the basis weight and the thickness of the mats are the governing factors in calculating solidity. Gelatin contains 8%–13% moisture and has a relative density of 1.3–1.4 g cm^−^^3^ [33], so the polymer density of gelatin was chosen as 1.35 g cm^−3^. Moreover, the basis weight was measured by taking the mean of the 5 samples that were cut out in 5 × 5 cm^2^. Because the samples could not be peeled off the spunbond support fabric, they were cut from 5 different places in the neat spunbond support fabric and weighed one by one and the mean weight was recorded. The net weight of the nanofiber coating was obtained by subtracting the mean weight of the neat support fabric from the mean weight of the nanofiber-coated support fabric.


(1) Solidity (α)=(Basis weight/(Thickness×Polymer Density))

## 4. Results and discussion

### 4.1. Morphology of the gelatin mats

**Figure 1:**
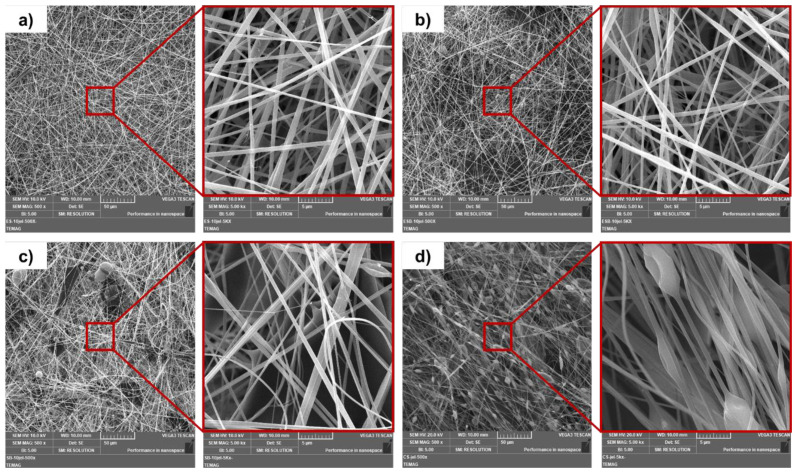
SEM images of 10 wt.% gelatin mats produced by different methods a) ES, b) EB, c) SB, and d) CS (for each sample, scale bars are 50 μm and 5 μm, respectively.).

**Figure 2:**
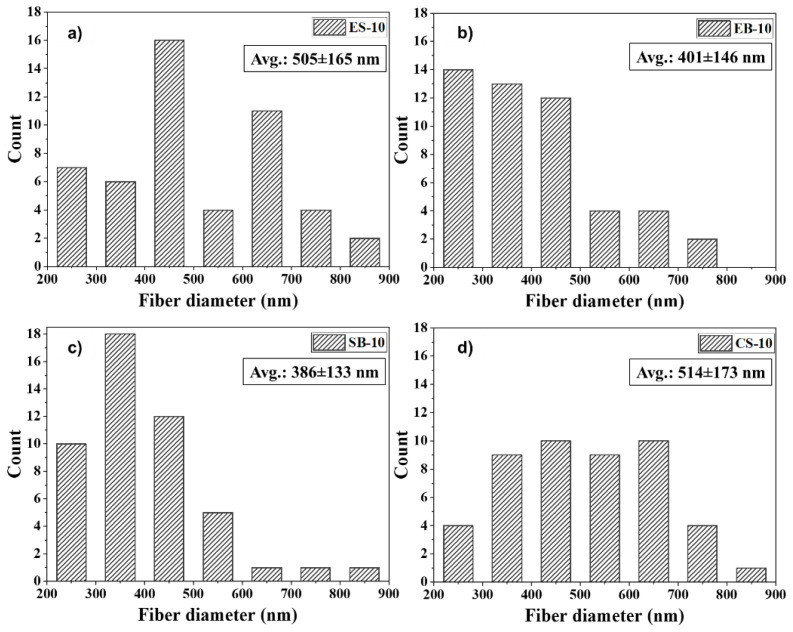
Fiber diameter distribution of 10 wt.% gelatin mats produced by different methods a) ES, b) EB, c) SB, and d) CS (for each sample, scale bars are 50 μm and 5 μm, respectively).

SEM images and the fiber diameter distribution of 10 wt.% gelatin mats produced by different methods are given in Figures 1 and 2, respectively. An examination of Figure 1 shows bead formation in the ES-10, EB-10, and CS-10 fibrous mats and bead plus droplets in the SB-10 fibrous mat. It is also seen that the ES-10 and EB-10 fibrous mats are composed of straight fibers. The SB-10 fibrous mat consists of relatively straight fibers, while the CS-10 sample consists of aligned but knotty fibers.

According to Figure 2, the thinnest fibers were produced by the SB, EB, ES, and CS methods, in that order. The fiber diameter distribution range, from wide to narrow, belongs to CS-10, ES-10, EB-10, and SB-10 fibrous mats, respectively.

**Figure 3:**
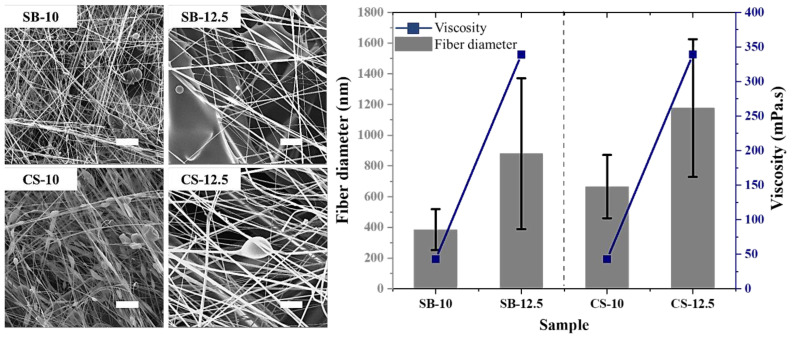
SEM images and fiber diameter–viscosity comparison graph of the SB and CS samples produced with 10 wt.% and 12.5 wt.% concentrated solutions (scale bar is 20 μm).

In Figure 3, SEM images and the fiber diameter–viscosity comparison graph of the SB and CS samples produced with 10 wt.% and 12.5 wt.% concentrated solutions are given. As seen in the SEM images, the fiber diameters increased almost 2-fold in both systems due to the increase in the concentration and the consequent increase in the viscosity. The viscosities of the 10 wt.% and 12.5 wt.% gelatin solutions are 43 mPa.s and 339 mPa.s, respectively. Although needles of the same diameter were used in production, the fibers produced in the CS system were thicker than those produced in the SB system for both concentrations.

**Figure 4:**
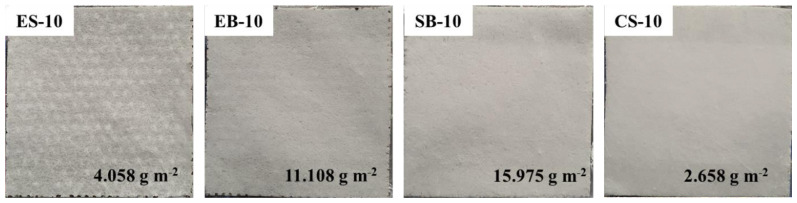
Digital camera photos and weights of the samples with 10 wt.% concentration.

There are perforated, metal, and cylindrical collectors in the nanofiber production methods used in the study. This, with the presence of electric fields (ES and EB methods), causes more loading at the points where the nonwoven fabric comes into contact with the collector and, therefore, more fiber is collected at these points. As a result, patterns are formed on the nonwoven fabric. Digital camera photos and weights of the samples with 10 wt.% concentration are shown in Figure 4.

### 4.2. Air permeability of the samples

Table 3 presents the fiber diameters, fabric thicknesses, basis weights, fabric porosities, and air permeabilities of all samples. According to the table, SB-10 and EB-10 mats not only have the thinnest fiber diameters, but also have the highest weights of 15.975 g m^−2^ and 11.108 g m^−2^, respectively. This suggests that the production of efficient fibers with minimal loss was achieved through these two production methods with 10 wt.% solution concentration compared to the other methods. Despite the fact that ES-10 mat, with the third thinnest fiber diameter, has a weight of 4.058 g m^−2^, it has the lowest air permeability (53 ± 4 mm s^−1^) due to the fibers being more attracted to each other and forming a more compact structure as a result of the electric field [24]. The effect of the electric field is more clearly evident when the values of the CS-10 mat are examined. Although the CS-10 mat has a similar fiber diameter value (514 nm) to the ES-10 mat, its weight is approximately two times lower, its fabric thickness is 1.5 times higher, and its air permeability value (692 mm s^−1^) is 13 times higher than that of the ES-10 mat. The lack of an electric field or pressurized air, which causes a more compact fabric structure in the ES, EB, or SB methods, results in a more fluffy fabric structure in the CS method. On the other hand, with increasing solution concentration from 10 wt.% to 12.5 wt.%, the air permeability of SB and CS mats increased due to the increasing fiber diameter. Another reason is that the number of fibers formed also decreased and the number of droplets increased. In particular, when SB-10 and SB-12.5 mats produced by the SB method are compared, the weight of the SB-12.15 mat (0.498 g m^−2^) decreased 32-fold.

### 4.3. Solidities of the samples

**Figure 5:**
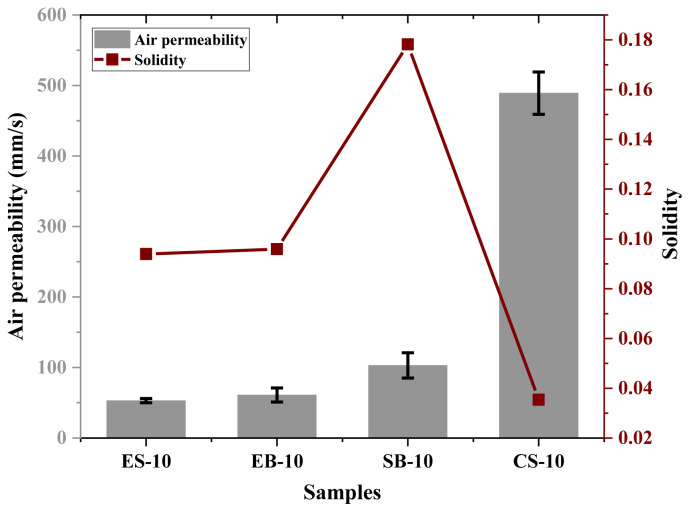
Comparative air permeability and solidity graphs of the samples with 10 wt.% concentration.

We can explain solidity as the solid volumes per area of the samples. Solidity graphs and comparative air permeability of the samples with 10 wt.% concentration are given in the Figure 5. When Figure 5 and Table 1 are examined together, it is seen that if the sample has low basis weight, it has lower solidity. On the other hand, if fabrics produced under equal conditions have high solidity, they are expected to have low air permeability [34]. However, although ES-10 and EB-10 fibrous mats had lower solidity values than the SB-10 fibrous mat, their air permeability was also lower. This situation is completely related to the existence of the electric field. The presence of the electric field causes the fibers to be more aligned [35] and so to have a denser structure.

## 5. Conclusion

In the present study, fibrous mats produced using gelatin solutions of different concentrations were analyzed and 4 different methods were compared.

- When using low-viscosity gelatin solution, the thinnest fiber production was possible with the SB system, while the straightest fibers were produced with the ES and EB methods due to the presence of the electric field. According to the fiber diameter distribution graphs, more uniform fibers are produced by compressed air in the SB and EB methods.
- With the increase in solution concentration, more fiber production is realized with the CS method, while the fiber formation was adversely affected in the SB method and more droplets were formed. This indicates that the fiber producible viscosity range is quite different, especially between SB and CS.
- Although the same amount of solution and the same solution concentration are used in production, the fibers’ ratio, morphology, and physical properties are different due to the differences between each method. Especially in the ES and EB methods, the electric field significantly increases fabric density. For this reason, solidity values are high but air permeability values are low in the samples.

According to the results, all methods have their respective advantages and disadvantages. We cannot say that one of them is better for medical applications, since successful gelatin fibers are produced by determining the appropriate parameters in each system. Further research is needed for a clear explanation. It is the case that all methods can be used in medical application areas depending on the product to be created. However, it is seen that the air permeability value, which is an important feature in wound healing, of the SB-12.5 sample, which is one of the nanofiber samples produced in this study, is much higher than that of the other production methods. However, due to the high fiber thickness and high droplet formation, this sample cannot form a high-quality wound dressing. At this point the CS-10 example is a good option, because, in addition to high fiber fineness and low sample thickness, it has a porous structure as the SB-12.5 sample and although its air permeability values are not as good as the SB-12.5 sample, it is higher than that of the other samples.

## Figures and Tables

**Table 1:** The advantages and disadvantages of the four nanofiber production methods.

Production methods	Advantages	Disadvantages
**ES**	Simple system [20]	Unsafe due to high voltage [20,21]
	Easy production [20]	Random fiber position [20]
	Large-scale production [20]	Requiring conductive solution [20]
	Narrow fiber diameter distribution [22]	Low production rate [21,23] limited both the commercial applicability of electrospun fibers and the capability of rapidly applying fibers [22]
**EB**	Easy production	High voltage
	Large-scale production [24,25]	More production parameters
	Large fiber diameter distribution but smaller than SB fibers	
	High production rate than ES	
**SB**	Simpler system than ES [22]	Less mechanical performance of fibers due to high pressure [21]
	Easy production	More droplet production with high viscous solutions
	Large-scale production [22,24]	
	Narrow fiber diameter distribution	
	High production rate than ES and EB	
	Safe production due to no electricity [23]	
**CS**	Easy production [17]	Higher bead production with low viscous solutions
	Obtaining free-standing and durable mats [17]	Obtaining thicker fibers than EB and SB fibers
	High production rate [17,18] than ES, EB, and SB	
	Low cost [17,19]	
	Safe production due to no electricity [15,17]	

**Table 2:** Production parameters used in all methods.

Production methods	Solution concentration (%)	Distance between the nozzle and the collector (cm)	Needle diameter (G)	Electric voltage (kV)	Solution feed rate (mL h^−1^)	Air pressure (bar)	Nozzle rotation speed (rpm)	Collector rotation speed (rpm)
**ES**	10	35	22	30	1	-	-	1000
**EB**	10			30	5	3	-	
**SB**	10–12.5			-	5	3	-	
**CS**	10–12.5			-	30	-	8000	

**Table 3:** All physical properties of all fibrous mats produced.

Samples	Fiber diameter (nm)	Thickness (mm)	Basis weight (g m^−2^)	Solidity (%)	Air permeability (mm s^−1^)
**ES-10**	505 ± 165	0.0320	4.058	9.39	53 ± 4
**EB-10**	401 ± 146	**0.0858**	11.108	9.59	61 ± 10
**SB-10**	386 ± 133	0.0664	**15.975**	17.82	103 ± 18
**SB-12.5**	880 ± 491	0.0125	0.498	2.95	**1080 ± 15**
**CS-10**	514 ± 173	0.0556	2.658	3.54	692 ± 24
**CS-12.5**	**1177 ± 448**	0.0650	2.051	**2.34**	706 ± 34
